# Red yeast rice induces less muscle fatigue symptom than simvastatin in dyslipidemic patients: a single center randomized pilot trial

**DOI:** 10.1186/s12872-017-0560-z

**Published:** 2017-05-18

**Authors:** Yangjing Xue, Luyuan Tao, Shaoze Wu, Guoqiang Wang, Lu Qian, Jiwu Li, Lianming Liao, Jifei Tang, Kangting Ji

**Affiliations:** 1 Department of Cardiology, The Second Affiliated Hospital and Yuying Children’s Hospital of Wenzhou Medical University, Xueyuanxi Road, No 109, Wenzhou, Zhejiang, 325000 China; 2 Department of Oncology, Academy of Integrative Medicine, Fujian University of Traditional Chinese Medicine, Huatuo Road, No 1, Fuzhou, Fujian 350122 China

**Keywords:** Muscle fatigue symptom, Physical activity, Red yeast rice, Simvastatin, Dyslipidemia, Lipid-lowering effects

## Abstract

**Background:**

About 10–15% patients who take statins experience skeletal muscle problems. Red yeast rice has a good safety profile could provide a compromise therapeutic strategy. Therefore, the aim of this study was to evaluate the effects of red yeast rice, when compared to simvastatin, on the muscle fatigue symptom and the serum lipid level in dyslipidemic patients with low to moderate cardiovascular risk.

**Methods:**

A total of 60 dyslipidemic patients with low to moderate cardiovascular risk were recruited and randomly assigned to receive either simvastatin (*n* = 33) or red yeast rice (*n* = 27) for 4 weeks. The muscle fatigue score, the physical activity, the serum lipid profile and the safety profile were then evaluated.

**Results:**

At the end of study, the fatigue score was significantly increased in patients treated with simvastatin, whereas no significant change was observed in patients receiving red yeast rice. In addition, the physical activity level was significantly decreased in patients from simvastatin group when compared to those from red yeast rice group. Similar lipid-lowering effects were observed in two groups. The safety profile was not affected after the treatments.

**Conclusions:**

Among dyslipidemic patients with low to moderate cardiovascular risk, red yeast rice induced less fatigue side effect and exerted comparable lipid-lowering effects when compared to simvastatin in this pilot primary prevention study.

**Trial registration:**

NCT01686451.

## Background

Statins have been shown to be beneficial for both primary and secondary cardiovascular (CV) prevention [1–5]. However, despite the efficacies of these agents in terms of lowering lipid levels, the rate of CV events, and, in some samples, mortality, some patients are unable to tolerate the adverse effects [6]. Unfortunately, failure to adhere to statin therapy can result in adverse CV outcomes [7, 8].

Increased fatigue has been recognized as one of the adverse effects of statins [9–13]. In a recent large scale randomized controlled trial, the majority of the patients were reported experiencing fatigue [13, 14]. Red yeast rice, which is a traditional dietary seasoning from the *MONASCUS purpureus* mold that contains lovastatin (Monacolin K) and other active ingredients, has been shown to exert lipid-lowering effects and CV benefits in both primary and secondary CV prevention studies [15–17]. Additionally, Red yeast rice is associated with few adverse events [15–17]. Unexpectedly, red yeast rice has been reported to have antifatigue effects [18].

In the present study, we compared the efficacy and fatigue-causing effects of red yeast rice and simvastatin in patients with dyslipidemia and moderate to low CV risk.

## Methods

### Participants

Patients with low-density lipoprotein-cholesterol (LDL-C) levels between 3 and 5 mmol/L (115–190 mg/dL) were enrolled. Patients were excluded if they were at a very high to high risk of fatal CV disease within 10 years based on the risk estimation system described in ESC/ESA guidelines (2011) of dyslipidemia management, [19] or they had symptomatic atherosclerotic disease (including coronary artery disease, peripheral arterial disease, and cerebrovascular disease), kidney failure or insufficiency, diabetes, a systematic coronary risk estimation (SCORE) value ≥5%, if they were currently using of any lipid-lowering medications or other medication, such as cyclosporin, erythromycin, clarithromycin, nefazodone, or any “azole” antifungal, (including fluconazole, itraconazole, ketoconazole, mibefradil, or protease inhibitors), or if they had conditions including active liver disease or unexplained persistently elevated transaminase levels, cancer, human immunodeficiency virus infection, a medical or psychiatric condition that prevented full study participation or follow-up (e.g., active psychosis), major surgery or hospitalization in the 3 months prior to study entry, if they were a female of childbearing potential, or if they were currently participating in another clinical trial.

The study was approved by the institutional review board, and all patients provided written informed consent.

### Randomization and masking

This trial was designed as a single-center, parallel-group study that took place at the medical clinic of the Second Affiliated Hospital of Wenzhou Medical University, China. According to the principle of the minimum distribution imbalance index, [20] the patients were randomly assigned to receive either red yeast rice (4 pills each containing 300 mg, hence in total 1200 mg daily) or simvastatin (0.5 pills each containing 40 mg, hence in total 20 mg daily) for 4 weeks. Patients were reminded weekly by telephone to take the medication on schedule and were asked to revisit at 28 ± 1 days (week 4).

### Study procedures

Fasting blood samples were collected at week 0 (randomization) and 4 (at end of study) for lipid profile analyses and clinical chemistry (including serum lipid concentrations, alanine transaminase (ALT), aspartate transaminase (AST), creatine phosphate kinase (CPK) and serum creatinine (Cr)). All clinical laboratory analyses were performed in the hospital’s central laboratory.

### Outcome measures

The primary endpoint was the fatigue score at the end of the study. Physical activity levels were also estimated. The baseline value was defined as the mean of the measurements obtained 1 week before randomization and on the day of randomization. The end-point value was defined as the measurement acquired after 4 weeks of treatment.

We estimated different aspects of fatigue levels with psychological and physical questionnaires. The fatigue scores were assessed with a fatigue questionnaire [21]. Changes in physical activity levels as evaluated with a short version of the international physical activity questionnaire [22] was also recorded. Because of drugs’ preparation issue, the measurement of the questionnaire was conducted in a single-blinded method that the physician did not know the treatment that patients received.

The predefined secondary efficacy endpoints included the percentage changes from baseline to the study endpoint in lipid parameters (triglyceride (TG), total cholesterol (TC), high-density lipoprotein-cholesterol (HDL-C), and LDL-C levels).

Safety was assessed by recording the prevalence and severity of adverse events and abnormal laboratory data. The patients who reported adverse events were also included.

Compliance was assessed at each visit by counting the number of returned tablets.

### Statistical analyses

All analyses were performed based on the intention-to-treat principle using data from all of the randomized patients. There were no treatment crossovers in the 4-week study period. Depending on the distributions, the continuous data are presented as medians (25th to 75th percentiles) or as the mean ± SD. The categorical data are presented as counts or proportions. The differences between groups were assessed with χ^2^ tests or Fisher’s exact tests for the categorical data and with the nonparametric Wilcoxon rank-sum test or Student’s *t* test for the continuous data. A 2-tailed value of *P* < 0.05 was considered to indicate statistical significance. All statistical analyses were performed using SPSS 17.0 software for Windows (SPSS for Windows version 17.0, Chicago, IL, USA).

## Results

### Patient characteristics

**Fig. 1:**
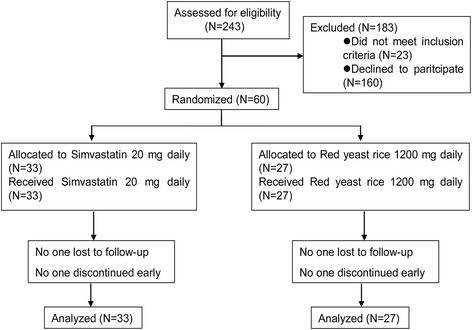
Trial flow chart

From August 10, 2012 to September 15, 2013, 243 patients were screened, and 60 patients who met the inclusion criteria were enrolled and randomly assigned to either the simvastatin (33 patients) or red yeast rice (27 patients) group. The flow of participants through the study is presented in Fig. 1. Table 1 shows the baseline characteristics of the patients, which were well balanced between the two treatment groups.

**Table 1:** Baseline characteristics of the patients^a^

Characteristic	Simvastatin (*n* = 33)	Zuezhikang (*n* = 27)	*P* value
Female sex, no. (%)	15 (45.5)	13 (48.2)	0.835
Age, yr	46.0 ± 7.0	47.0 ± 5.8	0.614
Current cigarette smoker, no. (%)	3 (9.1)	2 (7.4)	1.000
Arterial hypertension, no. (%)	26 (78.8)	16 (59.3)	0.101
Arterial blood pressure, mmHg			
Systolic	148 ± 19	148 ± 17	0.971
Diastolic	84 ± 7	83 ± 9	0.564
FBG, mmol/L	5.10 ± 0.36	5.33 ± 0.52	0.048
TC, mmol/L	5.91 ± 0.71	5.82 ± 0.73	0.488
TG, mmol/L	1.76 ± 1.00	1.61 ± 0.65	0.633
LDL-C, mmol/L	3.72 ± 0.48	3.74 ± 0.55	0.509
HDL-C, mmol/L	1.27 ± 0.25	1.23 ± 0.25	0.906
ALT, U/L	28.6 ± 10.6	30. 3 ± 13.3	0.570
AST, U/L	24.1 ± 7.0	25.6 ± 7.5	0.425
CPK, U/L	80.80 ± 24.90	91.7 ± 23.7	0.092
Cr, μmol/L	72.6 ± 14.4	75.6 ± 17.2	0.463
Fatigue score	19.6 ± 2.6	19.3 ± 1.9	0.592
Physical activity level			0.870
Low, no. (%)	14 (42.4)	3 (11.1)	
Moderate, no. (%)	10 (30.3)	12 (44.4)	
High, no. (%)	9 (27.3)	12 (44.4)	

^a^The plus-minus values are the means ± the SDs. The percentages do not sum to 100 due to rounding. *ALT* alanine transaminase, *AST* aspartate transaminase, *CPK* creatine phosphate kinase, *Cr* creatinine, *FBG* fasting blood glucose, *HDL* highdensity lipoprotein-cholesterol, *LDL* low-density lipoprotein-cholesterol, *TC* total cholesterol. *TG* triglyceride

### Efficacy results

**Fig. 2:**
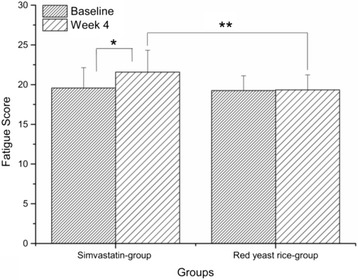
Comparison of the fatigue scores of the simvastatin and red yeast rice groups (mean ± SD). * *P* < .001 vs. baseline in the simvastatin group. ** *P* < .01 between the simvastatin and red yeast rice groups at week 4

Medication adherence was assessed by pill counting. All patients completed the self-rated fatigue assessment scale and the international physical activity questionnaire (short version) at the time of randomization and at week 4. The baseline values were comparable between the simvastatin and red yeast rice groups (Table 1). At week 4, the fatigue scores were significantly increased in the simvastatin group (*P* < .001 vs. baseline) and were significantly greater than those of the red yeast rice group (*P* < .01; Fig. 2). The fatigue scores did not change in the red yeast rice group (*P* = .16 vs. baseline; Fig. 2). Similarly, the physical activity levels, which were significantly reduced in the simvastatin group (*P* < .001 vs. baseline), remained unchanged in the red yeast rice group (*P* = .19 vs. baseline; Table 2) and were significantly lower in the simvastatin group than in the red yeast rice group (*P* < .001) at week 4 (Table 3).

**Table 2:** Comparison of the physical activity levels at baseline and at week 4

	Simvastatin (*n* = 33)		*P* value	Xuezhikang (*n* = 27)		*P* value
	Baseline	Week 4		Baseline	Week 4	
Physical activity level			<.001			0.19
Low, No. (%)	14 (42.4)	25 (75.8)		3 (11.1)	3 (11.1)	
Moderate, No. (%)	10 (30.3)	8 (24.2)		12 (44.4)	15 (55.6)	
High, No. (%)	9 (27.3)	0 (0.0)		12 (44.4)	9 (33.3)

**Table 3:** Physical activity levels of two treatment groups at week 4

	Simvastatin (*n* = 33)	Red yeast rice (*n* = 27)	*P* value
Physical activity level			<.001
Low, No. (%)	25 (75.8)	3 (11.1)	
Moderate, No. (%)	8 (24.2)	15 (55.6)	
High, No. (%)	0 (0.0)	9 (33.3)	

**Fig. 3:**
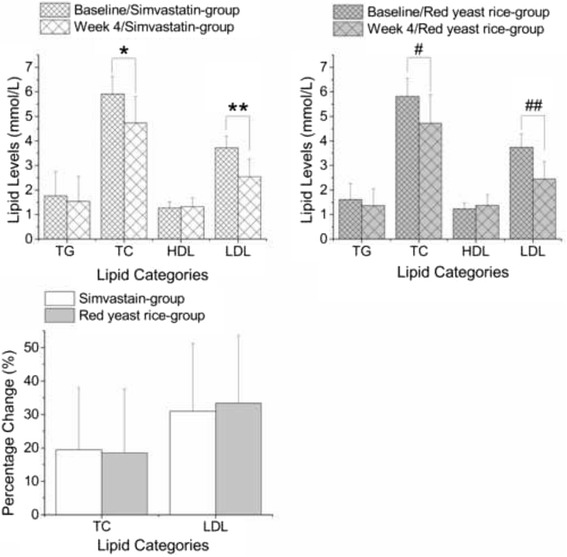
Comparison of the lipid-lowering efficacies of simvastatin and red yeast rice; **a** & **b** Comparison of lipid levels at week 4 and baseline in the simvastatin and red yeast rice groups (mean ± SD). No significant changes in TG or HDL levels were observed in either arm; **c** Comparison of the percentage changes in the lipid (TC and LDL) levels from baseline to week 4 (mean ± SD). The decrease in both the TC and LDL levels were comparable between the two groups.* TC levels, *P* < .001 vs. baseline in simvastatin group. ** LDL levels, *P* < .001 vs. baseline in simvastatin group. # TC levels, *P* < .001 vs. baseline in red yeast rice group. ## LDL levels, *P* < .001 vs. baseline in red yeast rice group

Before lipid-lowering treatment, there is no significant difference neither between the two groups of baseline level of TC(5.91 ± 0.71 vs. 5.82 ± 0.73 mmol/L for simvastatin and red yeast rice groups respectively, ns), nor between the two groups of baseline level of LDL-C(3.72 ± 0.48vs. 3.74 ± 0.55 mmol/L for simvastatin and red yeast rice groups respectively, ns). The administration of both simvastatin (20 mg daily) or red yeast rice (1200 mg daily) resulted in significant reductions in TC (−19.6% vs. -18.5% of baseline level for simvastatin and red yeast rice groups respectively, *P* < .001 vs. baseline for both) and LDL-C (−30.9% vs.-33.4% of baseline level for simvastatin and red yeast rice groups respectively, *P* < .001 vs. baseline for both) after 4 weeks of treatment (Fig. 3a-b) that were not significantly different between the two groups (*P* = 0.84 for the comparison of the percentage drop in the TC level and *P* = 0.64 for the comparison of the percentage drop in the LDL-C level; Fig. 3c). The improvements in TG and HDL-C concentrations were not significant in either treatment group (Fig. 3a-b).

### Safety results

**Fig. 4:**
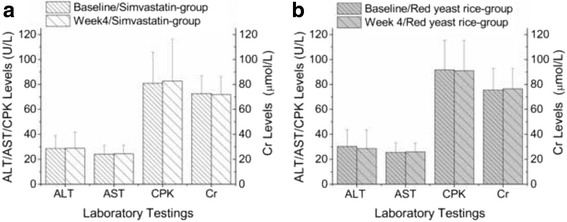
Comparison of the safeties at week 4 and at baseline (mean ± SD). No significant changes in ALT, AST, CPK or Cr levels were observed in either group

No significant increases of in the concentrations of ALT, AST, Cr, or CPK from baseline were observed in either arm (Fig. 4). No patient reported any adverse events.

## Discussion

Dealing with dyslipidemia should always be considered to be an essential and integral part of cardiovascular disease (CVD) prevention, and the tailoring of interventions to the baseline level of CVD risk is rational [19, 23]. In this trial, we compared the safeties and efficacies of simvastatin (20 mg daily) and red yeast rice (1200 mg daily) for patients with dyslipidemia and a 10-year risk of fatal CVD between 1% and 5% as evaluated with the SCORE system [19]. The findings indicated that the two treatments were comparable in terms of the decreases in LDL-C and TC levels. However, unlike the patients in the simvastatin group, the patients in the red yeast rice group did not experience fatigue or decreased physical activity.

Statins are generally well tolerated and rarely cause serious adverse events [1–5]. In the Cholesterol Treatment Trialist’s (CTT) meta-analyses that incorporated 26 randomized controlled trials, no increases in the risks for any non-CV cause of death and a small non-significant increase in rhabdomyolysis were observed in patients who were receiving statins [3]. Subsequent CTT studies and other meta-analyses that have addressed the issues of primary CV prevention have reported similar conclusions [1, 2, 4, 5].

However, statin-related fatigue has anecdotally been reported and confirmed by several studies, [9–14] including one randomized controlled trial [13] that enrolled 1016 participants (692 men and 324 women) who did not have heart disease or diabetes. The participants were randomly assigned to receive one of two statins (simvastatin or pravastatin) or placebo daily for six months. After six months, the participants taking the statins exhibited greater increases in overall fatigue than those who were taking the placebo. This effect was particularly pronounced in the women. The underlying mechanisms of statin-associated fatigue remain unclear but might be categorized as a manifestation of myopathy due to mitochondrial dysfunction [24–27]. Doctors should take these issues into account when considering the prescription of statins.

Although the potential benefits of prescribing statins to a patient who is at high risk of CV problems might outweigh the risks of side effects, such as increased levels of fatigue, the opposite might be true for a person who is at a low risk for CV problems. This balance should be decided on a patient-by-patient basis by the doctor and the patient. An alternative approach is the selection of medications other than statins. In the present study, we compared the safeties, specifically in terms of fatigue, and efficacies of simvastatin and red yeast rice in patients with dyslipidemia and moderate to low CVD risks. Red yeast rice is a Chinese herbal medication that has been approved by the China Food and Drug Administration for dyslipidemia. Red yeast rice contains a family of monacolin-related substances, one of which is a naturally occurring lovastatin. In a large-scale randomized, placebo-controlled trial, called the China Coronary Secondary Prevention Study, 4870 patients with prior myocardial infarctions and baseline cholesterol levels between 170 mg/dL-250 mg/dL (4.40–6.47 mmol/L) exhibited significantly reduced rates of CV events following red yeast rice treatment [16]. In a meta-analysis involving 9625 participants with primary hyperlipidemia, red yeast rice preparations appeared to be as effective as statins in lipid modification [15]. In these trials, [15] Xuezhikang (red yeast rice preparation) was used at dosage of 1.2 g/day (containing 10 mg of lovastatin), Zhibituo (red yeast rice preparation) at 3.15 g/day (containing 9 mg of lovastatin), simvastatin at 10–20 mg/day, pravastatin at 10 mg/day, lovastatin at 20 mg/day, atorvastatin 10 mg/day, and fluvastatin 20 mg/day. Moreover, in sharp contrast to simvastatin, we did not observe any significant effect of red yeast rice on fatigue scores or physical activity levels after 4 weeks of treatment. In the present study, we found that the lipid modification efficacies both of red yeast rice (at 1200 mg daily) and simvastatin (at 20 mg daily) were comparable. Although the beneficial changes were limited to the TC and LDL-C parameters, these changes were still highly important because the current guidelines recommend that LDL-C should be the primary target of therapy [19, 23].

There are some limitations to this research. First, the sample size was relatively small, and future studies with larger sample sizes are needed to validate our results. Secondly, we followed the participants for only 4 weeks; thus, the long-term outcomes remain to be determined. Thirdly, two possibilities might confound the effects of simvastatin and red yeast rice on fatigue score and physical activity level reported by the present pilot study: 1) small sample effects and potential baseline disparities in fatigue score, physical activity level, and hypertension; and 2) population characteristics that might place them at higher risk, e.g. Asian ethnicity coupled with a high fraction of hypertension.

## Conclusions

Red yeast rice had similar lipid lowering properties to simvastatin in this small, short-term primary prevention trial, and may cause less fatigue. Further study of red yeast rice’s long-term safety and efficacy in this patient population is warranted.

## Abbreviations

ALT: Alanine transaminase
AST: Aspartate transaminase
CPK: Creatine phosphate kinase
Cr: Serum creatinine
CTT: Cholesterol treatment trialist’s
CV: Cardiovascular
CVD: Cardiovascular disease
HDL-C: High-density lipoprotein-cholesterol
LDL-C: Low-density lipoprotein-cholesterol
SCORE: Systematic coronary risk estimation
TC: Total cholesterol
TG: Triglyceride
